# Psychiatric Disorders Mediate the Association between Floods and Dementia: A Prospective Cohort Study in the UK Biobank

**DOI:** 10.1021/envhealth.4c00241

**Published:** 2025-03-13

**Authors:** Yao Wu, Bo Wen, Danijela Gasevic, Rongbin Xu, Zhengyu Yang, Pei Yu, Yanming Liu, Guowei Zhou, Yan Zhang, Jiangning Song, Hong Liu, Shanshan Li, Yuming Guo

**Affiliations:** † Climate, Air Quality Research Unit, School of Public Health and Preventive Medicine, 2541 Monash University, Melbourne, VIC 3004, Australia; ‡ Centre for Global Health, Usher Institute, The University of Edinburgh, Teviot Place, Edinburgh EH8 9AG, United Kingdom; § Department of Dermatology, Xiangya Hospital, Central South University, Changsha, Hunan 410008, China; ∥ Monash Biomedicine Discovery Institute, Department of Biochemistry and Molecular Biology, 2541 Monash University, Melbourne, VIC 3800, Australia

**Keywords:** flood, psychiatric disorders, dementia, stress, depression

## Abstract

Flooding has become more frequent and severe worldwide, leading to an increased burden of psychiatric disorders (e.g., depression and anxiety). Psychiatric disorders are associated with an increased risk of subsequent dementia. However, the associations among floods, psychiatric disorders, and dementia are still unclear. Using a population cohort from the UK Biobank, we aimed to investigate the mediating role of psychiatric disorders on the associations between floods and dementia. In this study, cumulative exposure to floods over an eight-year period preceding the study baseline was assessed for each participant at residential addresses. Multivariable Cox proportional hazards regression was used to study the associations of flooding exposure with psychiatric disorders and dementia. Stratified analyses and mediation analyses were conducted to examine whether psychiatric disorders mediate the relationship between floods and dementia. During a median follow-up of 12.3 years (interquartile range: 11.6–13.0), 0.9% (2,028) of participants developed dementia and 9.5% (21,629) were diagnosed with psychiatric disorders. The flooding exposure was associated with an 8.0% increased risk of incident dementia (Hazard Ratio [HR]: 1.080, 95% CI: 1.023–1.141). The flood–dementia association was observed to be partially mediated by several subtypes of psychiatric disorders (overall proportion of mediation: 75.7%), with psychotic disorder accounting for 49.7% (indirect effect HR: 1.039, 95% confidence interval: 1.015–1.064) of flood-related dementia, followed by stress-related disorder (proportion of mediation: 18.1%), and depression (proportion of mediation: 3.9%). This study provides evidence of an increased risk of dementia associated with exposure to floods, with psychiatric disorders playing a crucial mediating role in the flood-related dementia pathway. These findings suggest that flooding exposure is a critical risk factor for dementia, and targeted interventions addressing postdisaster mental health may be crucial for dementia prevention in affected populations.

## Graphical abstract

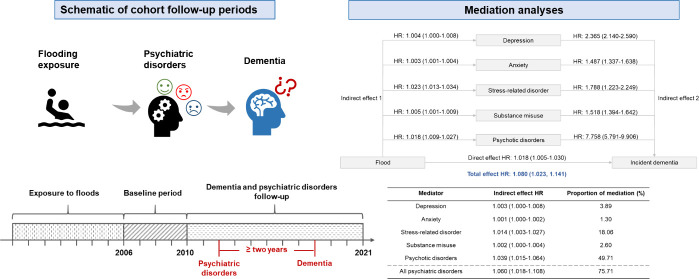

## 1 Introduction

Dementia is a major global health issue, with an estimated 58.7 million individuals living with dementia in 2020.^1^ This figure is projected to almost double every 20 years, reaching 152.8 million in 2050.^2^ The burden of dementia is substantial, resulting in 25.3 million disability-adjusted life years (DALYs) and 1.6 million deaths in 2019.^3^ Preventing or delaying the onset of dementia would substantially reduce this burden. While several modifiable risk factors (e.g., alcohol misuse, depression, social isolation) have been identified, a large portion of the risk of dementia remains unexplained.^4,5^

Floods are the most frequent type of natural disasters.^6^ The 21st century thus far has experienced 3,909 floods globally, accounting for 40.7% of all-natural disasters.^7^ In total, more than 1.8 billion people were affected by flood events, resulting in a direct loss of life of approximately 0.12 million people and causing injuries to over 0.33 million people.^7^ Experiencing natural disasters has been associated with an increased risk of dementia among older adults, with effects that tend to be long-lasting.^8–10^ For example, survivors of the 2011 Great East Japan Earthquake and Tsunami exhibited sustained cognitive decline over the subsequent decade.^9^ Accumulating evidence has shown that exposure to floods is also associated with an increased risk of dementia,^11,12^ as well as the risk factors of dementia, such as increased alcohol consumption,^13^ social isolation,^14,15^ and depression.^16^ For example, Yoshida et al. found that the older adults residing at home during the 2018 Japan Floods were at risk for cognitive decline and the number of antidementia medication users also increased within one year after the floods.^11,12^

Despite growing evidence linking flooding exposure to dementia, the mechanism is not well understood. One hypothesis is that psychiatric disorders may mediate this association, as studies have found a higher prevalence of psychiatric conditions among flood-affected individuals,^16–18^ potentially due to stressors such as injury, displacement, and financial loss.^19,20^ In addition, psychiatric disorders (e.g., depression, anxiety, post-traumatic stress disorders, psychotic disorders) have been associated with an increased risk of subsequent dementia.^21–24^ Proposed mechanisms for this link include unhealthy lifestyle behaviors and physiological changes induced by chronic stress.^25–28^ Given the close association between floods, psychiatric disorders and dementia, it is plausible to hypothesize that psychiatric disorders might contribute to the association between floods and dementia.

To our knowledge, no study has investigated the extent to which psychiatric disorders mediate the association between floods and dementia. To fill this gap, we used the UK Biobank project, a population-based cohort study with a large sample size, to comprehensively assess the effect of floods on psychiatric disorders and dementia, and the role of psychiatric disorders in the long term effects of flooding on the development of dementia.

## 2 Experimental Section

### 2.1 Study design and study population

The UK Biobank is a prospective cohort study that enrolled 0.5 million participants aged between 37 and 73 years through 21 assessment centers across England, Wales, and Scotland from 2006 to 2010. The cohort was followed up from baseline survey date until the study end date (December 31, 2021). We excluded participants lacking data on date of recruitment (*n* = 46), longitude and latitude data of residence (*n* = 11), those with dementia (*n* = 254) or psychiatric disorders (*n* = 75,107) at baseline, those with psychiatric disorders that occurred within a two-year period prior to the diagnosis of dementia (*n* = 1,003), and those with missing data on demographics, lifestyles, or socioeconomic status (*n* = 198,960). Comparison of baseline characteristics of study participants who were included and excluded due to missing values is presented in Table S1. Sensitivity analyses were also performed to check to robustness of our results with multiple imputations for the missing values. A total of 227,033 participants were included in the final analysis (Figure 1A). All participants in the UK Biobank study provided informed consent. The utilization of the data presented in this paper has been approved by the North West Multi-Centre Research Ethics Committee (06/MRE08/65).

**Figure 1:**
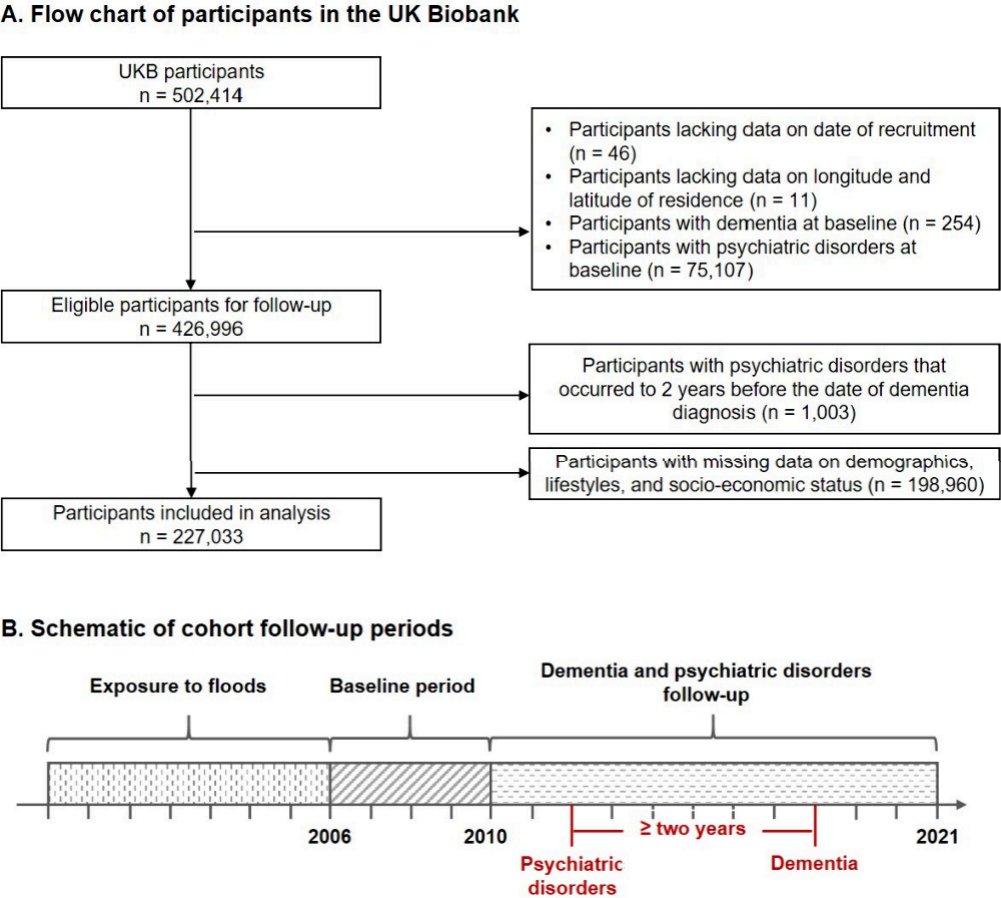
Flowchart of participants in the UK Biobank (A) and schematic of cohort follow-up periods (B). Exposure to floods: from 1-year to 8-year moving average preceding to baseline. Baseline period: participants were enrolled from 2006 to 2010. Dementia and psychiatric disorders follow-up: from baseline to the earliest date of incident dementia or the study end date (December 31, 2021). The diagnosis of psychiatric disorders should precede the diagnosis of dementia by a temporal interval of at least two years.

To explore the potential mediating role of psychiatric disorders, it was necessary to establish a temporal sequence where the exposure preceded the mediator, and the mediator preceded the outcome. To approach a chain of distinct events, we defined the flood exposure windows as diverse temporal intervals ranging from the current year (Lag 0) to seven years (Lag 0–7) preceding the baseline assessment. Psychiatric disorder diagnoses were then tracked following flood exposure, ensuring that their onset occurred after the exposure period. To further establish temporal precedence, dementia diagnoses were required to occur at least two years after the first recorded diagnosis of a psychiatric disorder. This time lag was chosen to reduce the risk of reverse causality, where early neurodegenerative symptoms might influence psychiatric conditions rather than the other way around.^29^ Additionally, we conducted sensitivity analyses using alternative time intervals between psychiatric disorder onset and dementia diagnosis to assess the robustness of our findings. By implementing these measures, we aimed to minimize potential biases related to timing and ensure a rigorous evaluation of the mediating role of psychiatric disorders in the relationship between flood exposure and dementia (Figure 1B).

### 2.2 Exposure assessment

We collected flood data from 1999 to 2010 (according to the baseline period of the UK Biobank) using the Dartmouth Flood Observatory (DFO), a comprehensive global catalog that documents flood events reported in news, governmental sources, and the FloodList (http://floodlist.com/) since 1985. For each flood event, we extracted detailed information, including start and end dates, centroids, affected geographic areas, and severity ratings. Flood severity was determined based on the estimated recurrence interval and overall impact. Specifically, class 1 represents large flood events with recurrence intervals of 1–2 decades; class 1.5 denotes very large events with recurrence intervals exceeding 2 decades but less than 100 years; and class 2 indicates extreme events with recurrence intervals over 100 years (Table S2). Numerical values of 1, 1.5, and 2 were assigned to these classes to create an ordinal scale, with higher values reflecting greater severity and potential impact. Rather than relying solely on strict physical measurements, this method integrates expert judgment, media reports, and satellite observations, effectively approximating the decimal logarithm of the mean return period. This provides a standardized framework for comparing the rarity and potential impact of flood events across different regions and time periods. Participants residing in areas affected by floods were considered exposed based on their home addresses. Drawing on existing studies,^30,31^ we calculated the yearly cumulative flooding exposure (i.e., flood index) for each participant, which accounts for both the duration (in days) and the severity of each flood event, using the following eq 1:

1 $${\mathrm{Flood index}}_{i,\mathrm{year}=m}=\overset{n}{\underset{j=1}{\sum }}{\mathrm{Duration}}_{ij}\times {\mathrm{Severity}}_{ij},\left(j=1,...,n\right)$$

where Flood index_*i*,year=*m*_ stands for the flood index in year *m* for participant *i*. Duration_*ij*_ and Severity_*ij*_ represent the duration and the severity of the *j*th flood event in year *m*, respectively. If no flood events occurred during a specific year, the flood index was set to 0.

To capture the long-term exposure to floods, we calculated the moving average of flooding exposure over different exposure windows (from 1-year to 8-year moving average) preceding the baseline.

### 2.3 Outcome and mediator assessments

The identification of the date and diagnosis for dementia and psychiatric disorders was accomplished by using record linkage to primary care data, hospital inpatient records, death register records, and self-reported medical conditions. Diagnoses from primary care, hospital inpatient, and death register records were mapped to 3-character International Classification of Diseases, 10th Revision (ICD-10) codes. Dementia was identified using the ICD-10 codes: F00 (dementia in Alzheimer’s disease), F01 (vascular dementia), F02 (dementia in other diseases), F03 (unspecified dementia), or G30 (Alzheimer’s disease). Psychiatric disorders were classified as follows: depression, F32–F33; anxiety, F40–F41; stress-related disorder, F43; substance misuse, F10–F19; and psychotic disorders, F20–F29. In addition to clinician-recorded diagnoses, self-reported health conditions were collected via a touchscreen questionnaire and subsequently reviewed during a verbal interview with a nurse. The date of each outcome was recorded using the earliest available information from self-reports, inpatient hospital records, primary care data, or death records.

### 2.4 Covariates

We considered the following covariates collected during the baseline survey based on prior evidence: age, sex, ethnicity, body mass index (BMI), educational attainment, average total annual household income before tax, cigarette smoking, alcohol consumption, physical activity, healthy diet score, Townsend deprivation index (TDI), and assessment centers.^4,32^ These covariates were considered either risk factors of dementia or moderators of flood–dementia associations based on our preliminary analyses. BMI was calculated from objectively measured weight and height as weight over height squared (kg/m^2^). Educational attainment was coded in four categories: college or university degree, A/AS levels or equivalent, O levels/GCSEs or equivalent, or none of the above. Smoking status was categorized into three groups: current, former, and never. We defined moderate alcohol consumption (also known as low-risk alcohol consumption) as consuming no more than one drink/day for women and two drinks/day for men. In the UK, one drink is measured as containing 8 g of ethanol.^33^ Annual household income before tax was categorized into five groups: < £18,000, £18,000–£30,999, £31,000–£51,999, £52,000–£100,000, and > £100,000. Physical activity was assessed using the International Physical Activity Questionnaire-Short Form (IPAQ-SF).^34^ Participants were categorized as having ‘high’ (≥1,500 metabolic equivalent [MET]-minutes/week), ‘moderate’ (≥600 MET-minutes/week), or ‘low’ levels of physical activity. Healthy diet score was calculated based on several dietary factors, including vegetable intake (≥3 servings/day), fruit intake (≥3 servings/day), whole grains (≥3 servings/day), refined grains (≤1.5 servings/day), fish intake (≥2 servings/day), unprocessed red meat intake (≤2 servings/week), and processed meat intake (≤2 servings/week). Each favorable dietary factor was assigned one point, and a healthy diet was defined as a diet score of ≥4. TDI was used to determine the level of area deprivation. Participants were classified as either high (TDI above the median) or low.^35^

### 2.5 Statistical analysis

Cox proportional hazards regression models were used to estimate the risk of incident dementia associated with floods, inspecting the flooding exposure with various exposure windows (from 1-year to 8-year moving average) to identify the optimal exposure windows. Survival time for each participant was calculated as the duration from the baseline through the end of the follow-up. The proportional hazards assumption was tested based on the scaled Schoenfeld residuals. The violation of the proportional hazards assumption was solved by treating the assessment center as a strata in the models. A two-stage analysis was applied to explore the role of psychiatric disorders in the association between floods and incident dementia. In the first stage, we stratified the analyses by psychiatric disorders to explore the potential modifying effect of psychiatric disorders. In the second stage, we conducted mediation analyses to explore the mediation effect of psychiatric disorders. Structural equation models were used to decompose the total effect (TE) of floods on incident dementia into its direct (DE) and indirect effect (IE) through psychiatric disorders.^36,37^ TE, DE, IE, and their 95% CIs were computed using bootstrapping procedures (1000 replications). Results are presented as hazard ratios (HRs) and their 95% confidence intervals (95% CIs). Finally, we explored the potential nonlinear relationship between flooding exposure and incident dementia, stratified by psychiatric disorders, by modeling floods using natural cubic splines with three degrees of freedom.

### 2.6 Sensitivity analysis

We carried out the following sensitivity analyses: (1) we further adjusted for mean temperature and relative humidity with a natural cubic spline with three degrees of freedom. Temperature and relative humidity data were downloaded from the European Centre for Medium-Range Weather Forecasts Reanalysis v5 (ERA-5) reanalysis data set with a spatial resolution of 0.1° × 0.1°. (2) We further adjusted for the history of other chronic diseases including hypertension, diabetes mellitus, cardiovascular diseases, and chronic obstructive pulmonary disease. (3) We repeated our analyses by restricting participants living in the current address for at least ten years. (4) Multiple imputations were used by chained equations for the missing values. Five imputed data sets were created, and their results were combined using Rubin’s rules.^38^ (5) We further adjusted for apolipoprotein E (APOE) ε4 status. APOE genotypes (ε2, ε3, and ε4) were ascertained based on the haplotypes of rs429358 and rs7412 (T-T, T-C, and C-C, respectively). Based on the APOE genotypes, we categorized participants into three risk groups: high APOE risk (ε2ε4, ε3ε4 or ε4ε4 genotypes), intermediate risk (ε3ε3 genotype), and low risk (ε2ε2 or ε2ε3 genotypes).^39,40^ (6) To ensure the robustness of our results from the mediation analysis, we conducted sensitivity analyses by excluding psychiatric disorders that occurred within one or three years before the onset of dementia. (7) We further adjusted for the social isolation in the model.

## 3 Results

This study included 227,033 participants, of whom 0.9% (2,028) developed dementia. Approximately 9.5% (21,629) were diagnosed with psychiatric disorders. The median follow-up time was 12.3 years (interquartile range [IQR]: 11.6–13.0). At baseline, the mean (standard deviation [SD]) age was 56.1 (8.1) years. 53.8% were male and 97.2% were White European (Table 1). From 1998 to 2010, a total of 37 flood events were recorded across the UK, with the mean duration of 3.8 days (ranging from 1 to 16 days). Most of flood events were caused by heavy rain. The mean (SD) level for the flood index in the baseline year was 16.8 (19.5) among participants without incident dementia and 18.7 (20.1) among those with dementia; in the eight years preceding the baseline, the corresponding value was 5.5 (5.1) for participants without incident dementia and 5.5 (5.0) among those with dementia (Table S3). The spatial distribution of the flooding exposure across participants is shown in Figure S1.

**Table 1:** Baseline Characteristics of Study Participants, Stratified by Psychiatric Disorders and Dementia Status

		No dementia, Psychiatric disorders		With dementia, Psychiatric disorders	
	Overall	Absent	Present	Absent	Present
*N*	227,033	203,681	21,324	1,723	305
Age, mean (SD)	56.1 (8.1)	56.0 (8.0)	56.0 (8.2)	64.6 (4.3)	62.9 (5.8)
Male, *n* (%)	122,171 (53.8)	109,229 (53.6)	11,556 (54.2)	1,166 (67.7)	220 (72.1)
White ethnicity, *n* (%)	220,749 (97.2)	198,092 (97.3)	20,668 (96.9)	1,687 (97.9)	302 (99.0)
BMI, mean (SD)	26.9 (4.3)	26.9 (4.3)	27.5 (4.6)	27.0 (3.9)	27.4 (4.5)
Household income, *n* (%)					
<£18,000	35,077 (15.5)	29,321 (14.4)	5,073 (23.8)	559 (32.4)	124 (40.7)
£18,000–30,999	53,853 (23.7)	47,657 (23.4)	5,520 (25.9)	583 (33.8)	93 (30.5)
£31,000–51,999	63,537 (28.0)	57,443 (28.2)	5,682 (26.6)	359 (20.8)	53 (17.4)
£52,000–100,000	57,423 (25.3)	53,021 (26.0)	4,189 (19.6)	188 (10.9)	25 (8.2)
>£100,000	17,143 (7.6)	16,239 (8.0)	860 (4.0)	34 (2.0)	10 (3.3)
Education attainment, *n* (%)					
College or university degree	24,839 (10.9)	21,012 (10.3)	3333 (15.6)	407 (23.6)	87 (28.5)
A/AS levels or equivalent	35,078 (15.5)	30,975 (15.2)	3797 (17.8)	259 (15.0)	47 (15.4)
O levels/GCSEs or equivalent	75,679 (33.3)	67,527 (33.2)	7508 (35.2)	554 (32.2)	90 (29.5)
None of the above	91,437 (40.3)	84,167 (41.3)	6686 (31.4)	503 (29.2)	81 (26.6)
Smoking, *n* (%)					
Never	124,148 (54.7)	115,688 (56.8)	7578 (35.5)	794 (46.1)	88 (28.9)
Previous	84,744 (37.3)	76,199 (37.4)	7562 (35.5)	851 (49.4)	132 (43.3)
Current	18,141 (8.0)	11,794 (5.8)	6184 (29.0)	78 (4.5)	85 (27.9)
Nonmoderate alcohol consumer, *n* (%)	179,918 (79.2)	160,551 (78.8)	17902 (84.0)	1209 (70.2)	256 (83.9)
Healthy diet score, mean (SD)	0.6 (0.5)	0.6 (0.5)	0.5 (0.5)	0.6 (0.5)	0.6 (0.5)
Physical activity, *n* (%)					
Low	38,935 (17.1)	34,387 (16.9)	4,208 (19.7)	280 (16.3)	60 (19.7)
Middle	58691 (25.9)	52859 (26.0)	5297 (24.8)	453 (26.3)	82 (26.9)
High	129407 (57.0)	116435 (57.2)	11819 (55.4)	990 (57.5)	163 (53.4)
High Townsend deprivation index, *n* (%)	103042 (45.4)	90772 (44.6)	11324 (53.1)	780 (45.3)	166 (54.4)

Figure 2 shows the results of models incorporating a natural cubic spline of flood index to estimate the HRs of incident dementia with floods, stratified by psychiatric disorders. For participants without psychiatric disorders, the exposure-response curve exhibits a positive and approximately linear pattern, with no apparent thresholds. Compared to those without psychiatric disorders, there was a steeper increase in the risk of incident dementia with increasing floods among those with psychiatric disorders, despite a slight decline initially.

**Figure 2:**
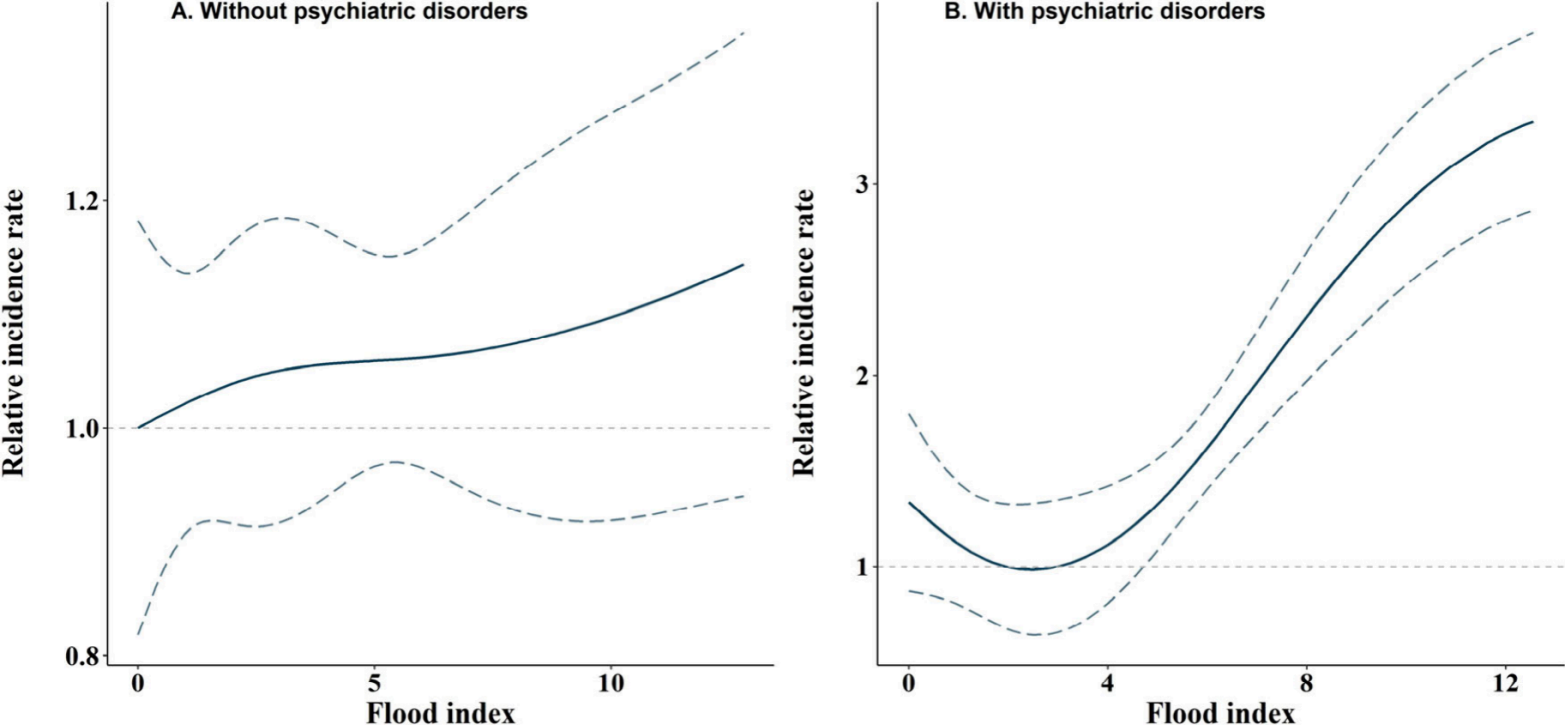
Exposure–response curve between floods (Lag 0–7) and incident dementia, stratified by psychiatric disorders. Floods are modeled using natural cubic splines with three degrees of freedom. The reference group is considered the exposure level with a minimum relative incidence rate. HR less than 1 denotes a smaller effect estimate than the effect of mean exposure level.

Figure 3 illustrates the estimated HRs of incident dementia with per unit increase in flood index, stratified by psychiatric disorder status (absent/present). Overall, we observed a higher risk of incident dementia with increasing exposure to floods among participants with psychiatric disorders, in comparison to those without psychiatric disorders. In the baseline year (Lag 0), the HRs were 0.997 (95% CI: 0.992–1.002) for participants without psychiatric disorders and 1.000 (0.988–1.012) for those with psychiatric disorders. When the exposure window expanded to 8 years preceding the baseline (Lag 0–7), the corresponding HRs increased to 1.011 (95% CI: 0.987–1.036) for individuals without psychiatric disorders and 1.070 (95% CI: 1.010–1.133) for individuals with psychiatric disorders. A similar pattern was found for subtypes of psychiatric disorders, except for anxiety (Figures S2–6 **)**.

**Figure 3:**
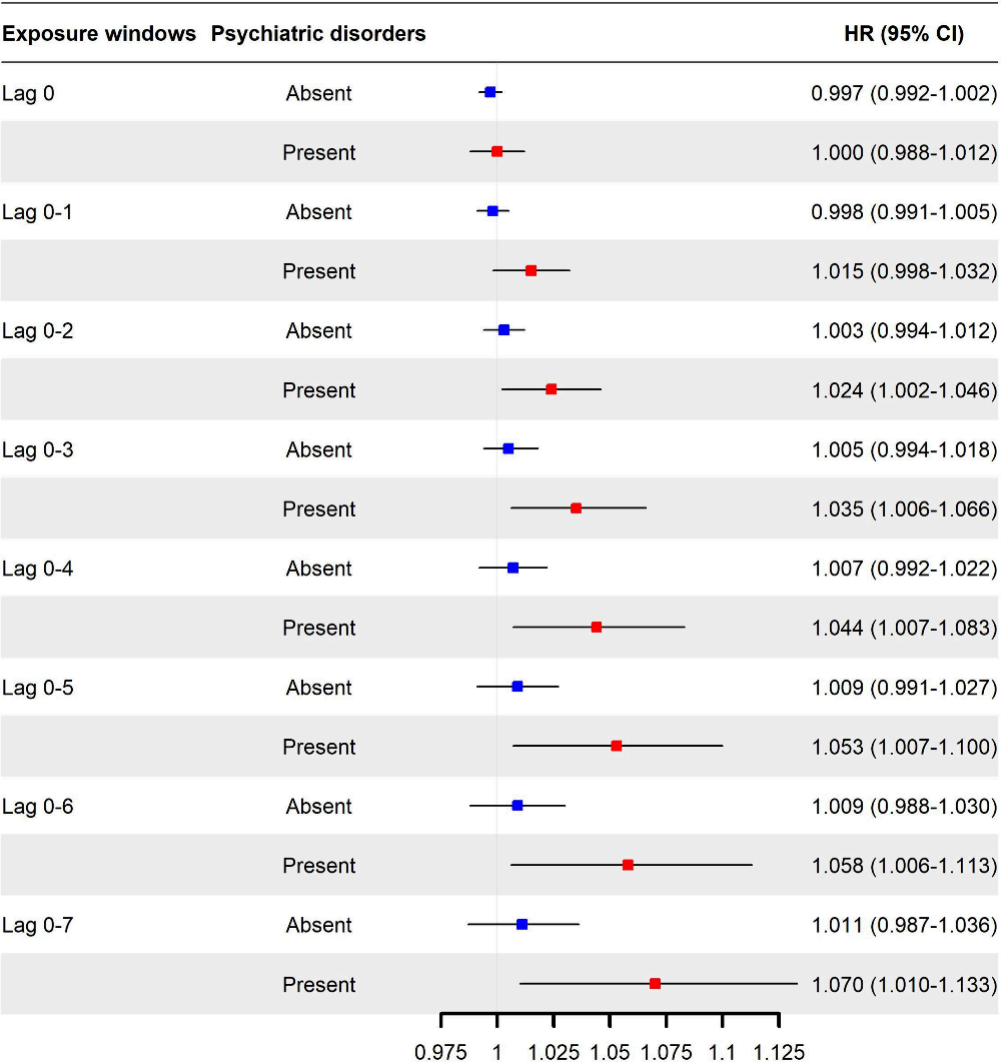
Associations between incident dementia and floods, stratified by psychiatric disorders. Estimates are hazard ratios associated with per unit increase in flood index derived from Cox proportional hazards regression models. Models are adjusted for age, body mass index, sex, ethnicity, annual household income before tax, educational attainment, smoking status, drinking status, physical activity, healthy diet, Townsend deprivation index, and assessment center.

Figure 4 shows the HRs for the associations between floods and both psychiatric disorders and incident dementia. Results indicated that the magnitude of associations with incident dementia after adjusting for psychiatric disorders increased as the exposure window expanded, with the highest value observed for lag 0–7 (the eight-year moving average preceding the baseline). The strongest association with all psychiatric disorders, as well as their specific subtypes (anxiety and psychotic disorders), was observed for the exposure windows less than two years preceding the baseline. In contrast, stress-related disorder and substance misuse exhibited the strongest association for lag 0–6 or lag 0–7. Considering its causal effect, we selected the exposure window with the strongest association for each outcome in subsequent mediation analysis.

**Figure 4:**
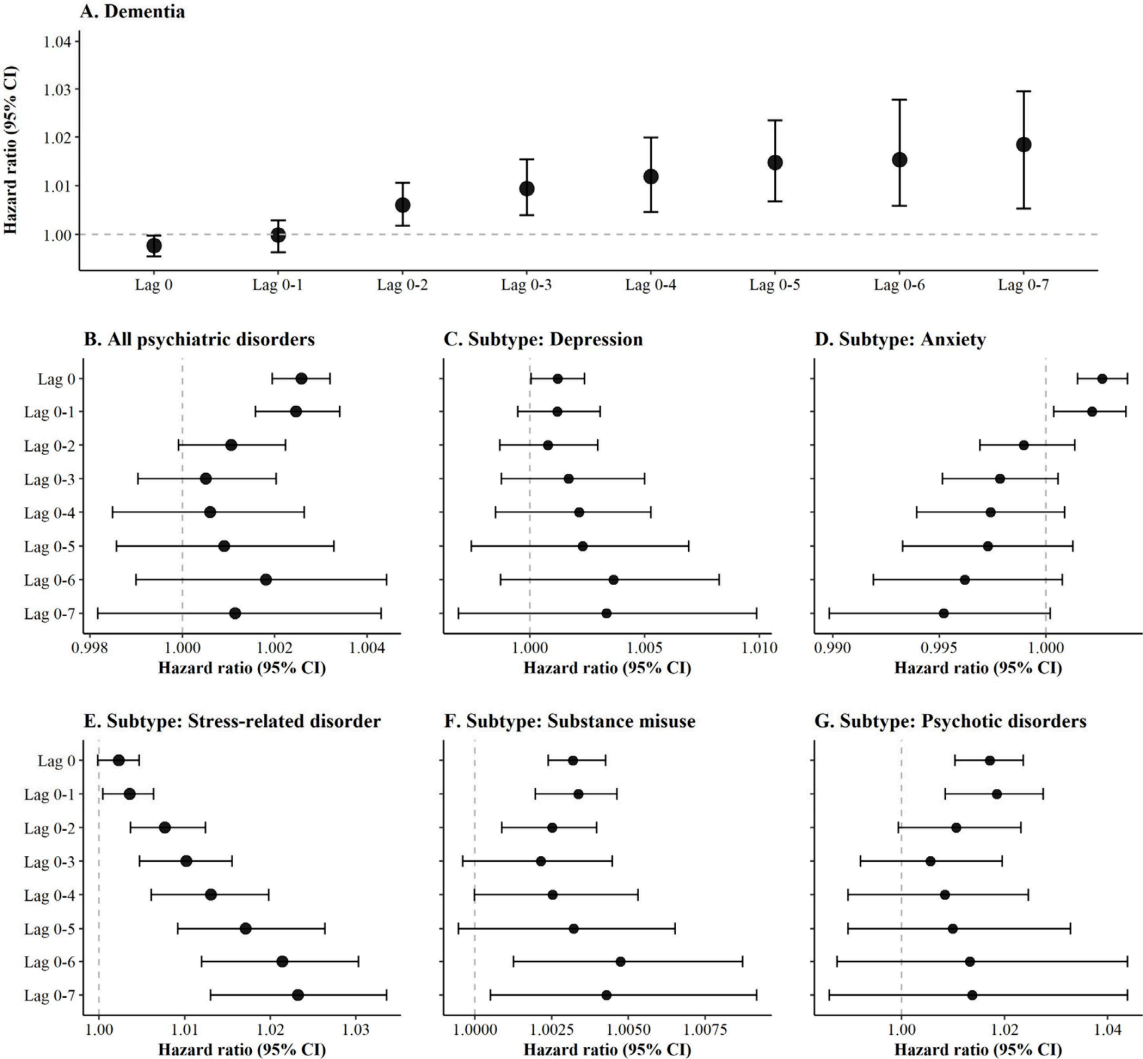
Associations of floods with incident dementia (A), psychiatric disorders (B), and its subtypes (C–G). Estimates are hazard ratios associated with per unit increase in flood index derived from Cox proportional hazards regression models. Models for psychiatric disorders (B) and their subtypes (C−G) are adjusted for age, body mass index, sex, ethnicity, annual household income before tax, educational attainment, smoking status, drinking status, physical activity, healthy diet, Townsend deprivation index, and assessment center. Models for dementia (A) are additionally adjusted for all subtypes of psychiatric disorders to capture the direct effect of floods on incident dementia.

Figure 5 shows the bootstrap results regarding the total, direct, and indirect effects of floods on incident dementia through psychiatric disorders. We observed a statistically significant direct effect of flooding exposure on incident dementia, with a direct effect HR of 1.018 (95% CI: 1.005–1.030). The effect of floods on dementia appeared to be partially mediated through psychiatric disorders, with an indirect effect HR of 1.060 (95% CI: 1.018–1.108) for all psychiatric disorders. This corresponds to approximately 76% of the total effect of floods on dementia. As a result, per unit increase in flood index was observed to be directly and indirectly associated with an increase of 8.0% in the risk of incident dementia (total effect HR: 1.080, 95% CI: 1.023–1.141). Among various specific subtypes of psychiatric disorders, psychotic disorder accounted for the largest proportion of mediation (proportion of mediation: 49.71%; indirect effect HR: 1.039, 95% CI: 1.015–1.064) in the association between floods and dementia, followed by stress-related disorder (proportion of mediation: 18.06%; indirect effect HR: 1.014, 95% CI: 1.003–1.027), and depression (proportion of mediation: 3.89%; indirect effect HR: 1.003, 95% CI: 1.000–1.008).

**Figure 5:**
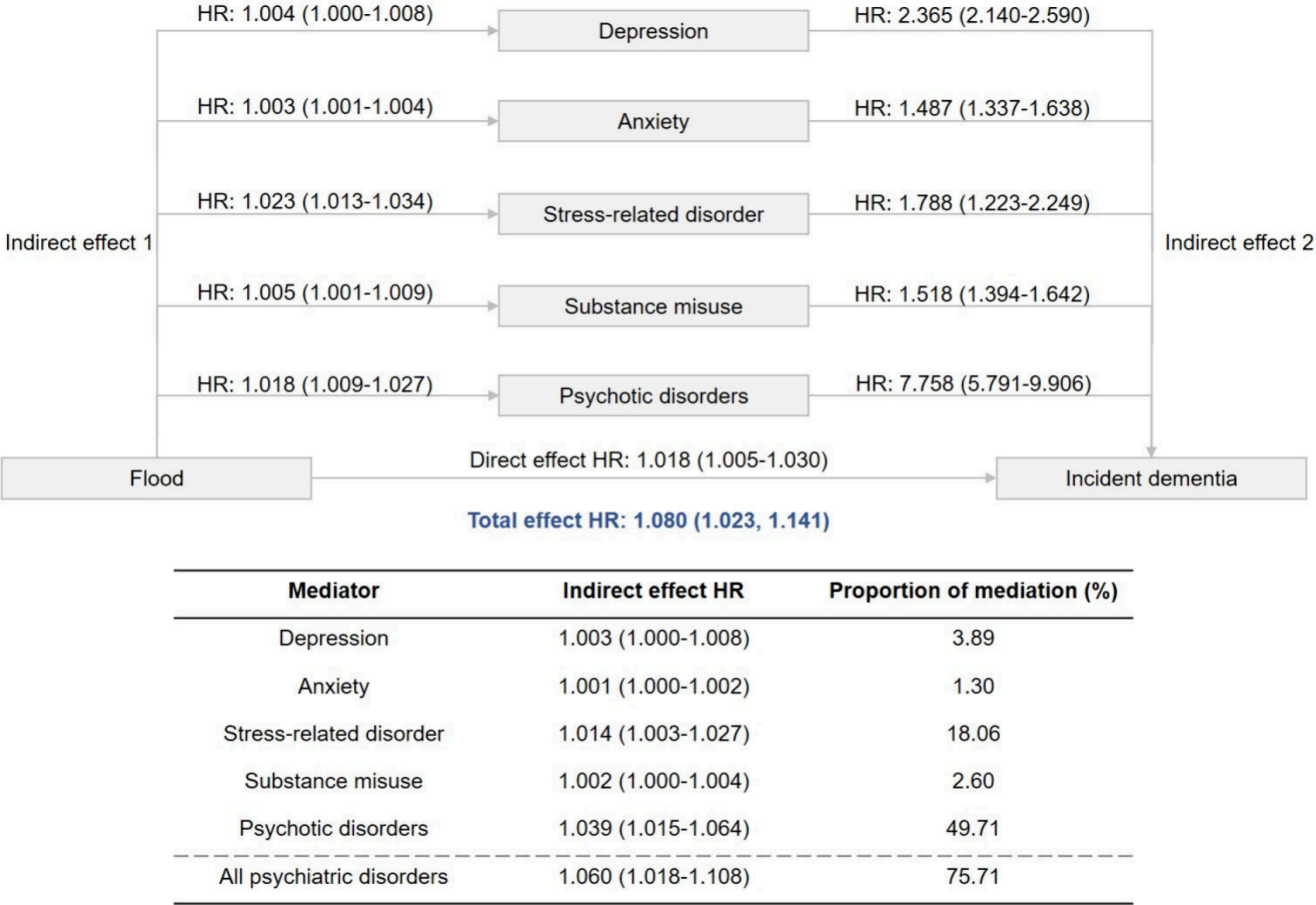
Bootstrap results for total, direct, and indirect effects of floods on incident dementia through psychiatric disorders. Estimates are hazard ratios associated with per unit increase in flood index derived from Cox proportional hazards regression models. 95% confidence intervals were computed using bootstrapping procedures (1000 replications). Models are adjusted for age, body mass index, sex, ethnicity, annual household income before tax, educational attainment, smoking status, drinking status, physical activity, healthy diet, Townsend deprivation index, and assessment center. IE = IE1 × IE2. TE = IE + DE. Abbreviations: IE, indirect effect. DE, direct effect. TE, total effect.

Our results remained consistent and robust with sensitivity analyses of further adjustment for mean temperature and relative humidity; further adjustment for the history of other chronic diseases; restricting analyses to participants living in the same location for at least ten years; complete case analyses after multiple imputations; using varying time intervals between the mediator and outcome; further adjustment for the APOE status; and further adjustment for social isolation (Tables S4–10).

## 4 Discussion

In our study with over 200,000 participants from the prospective UK Biobank cohort, we observed that exposure to floods was significantly associated with an increased risk of both dementia and all subtypes of psychiatric disorders. Specifically, the association between floods and dementia persisted for a duration of up to eight years, whereas the association between floods and psychiatric disorders was primarily observed within a two-year time frame following exposure. Moreover, our findings indicate that psychiatric disorders partially mediated the association between floods and dementia. The direct effect of floods on the risk of dementia, independent of psychiatric disorders, was also observed.

To date, no study has investigated the role of psychiatric disorders in the association between floods and dementia, although previous speculation in studies on floods and dementia/cognitive decline suggested that this association might be attributed to psychiatric disorders.^11^ Only one study, involving 3,566 survivors of the 2011 Great East Japan Earthquake and Tsunami, reported a stronger association between property damage and cognitive decline among individuals with depression,^41^ which aligns with our findings from stratification analyses. Our study extends this existing evidence by further testing the hypotheses of mediation and decomposing the direct and indirect effects of floods through psychiatric disorders. Notably, we observed that the strongest risk of dementia associated with floods occurred within an exposure window of eight years (Lag 0–7). In contrast, the risk of psychiatric disorders, as well as their specific subtypes (anxiety and psychotic disorders) related to floods was only significant for exposure windows of up to two years. This disparity in exposure windows suggests that the onset of anxiety and psychotic disorders are more likely to precede the occurrence of dementia following exposure to floods. This finding aligns with previous studies demonstrating that exposure to disasters like floods may lead to acute stress response within two years,^42–44^ with a decreasing trend over time. For example, the prevalence of depression and anxiety among individuals affected by the widespread flooding in the UK decreased from 19.1% and 30.6% to 12.2% and 15.7%, respectively, within two years.^42^ By contrast, exposure to floods and other disasters is associated with both short-term and long-term cognition decline, especially among older adults.^11,45^ This may account for the prolonged lag effects of floods on the onset of dementia, further suggesting that psychiatric disorders may mediate the relationship between flooding and dementia.

The biological mechanisms underlying the mediation effect of psychiatric disorders on the risk of dementia are not well understood; however, several plausible pathways have been proposed. Disaster experiences can cause a series of primary and secondary stressors. Primary stressors include trauma, injury, property damage, or the loss of a loved one.^46^ Secondary stressors relate to stressors that persist over a long time and/or have a long-term impact, for example, extreme physical conditions (e.g., living in an evacuation center) and psychological adversity (e.g., lack of social contact and personal privacy in the evacuation center) due to displacement, financial losses, unemployment, income loss, difficulties with insurance and compensation, and health-related concerns arising from limited access to health facilities and interruptions in treatment.^19,47^ These flood-related stressors have been linked to an increased risk of psychiatric disorders,^19,20^ which themselves serve as risk factors for dementia.^24,48^ Several pathways have been proposed to link psychiatric disorders to dementia,^24^ including behavioral changes (e.g., smoking, alcohol misuse, physical inactivity, and social isolation); vascular disease; excessive cortisol secretion;^49^ alterations in hippocampal function and structure;^25–28^ the increasing presence of neuritic plaques and neurofibrillary tangles;^50^ increased release pro-inflammatory cytokines;^49^ and decreased levels and activities of neurotrophic factors.^51^ For example, chronic stress has been shown to decrease the volume of gray matter in the right hippocampus,^27^ leading to impairment of cognitive functions.^28^ Anxiety and depression are associated with decreased densities of dendrites and spines in cornu ammonis area 3 of the hippocampus,^25^ further impairing learning and memory via mechanisms that disrupt the integrity of hippocampal dendritic spines.^26^

In this study, we observed that psychotic disorders exhibit a disproportionately high mediation effect in the association between floods and dementia, compared to other psychiatric disorders like depression or anxiety. While the risk of developing psychotic disorders following flood exposure was slightly higher, the stronger mediation effect was primarily driven by the greater effect size of psychotic disorders on dementia (Figure 5). This aligns with previous studies indicating a stronger link between psychotic disorders and increased dementia risk.^52–54^ For example, it was observed that nonaffective psychotic disorders were associated with increased dementia risk (Relative risk [RR] = 2.52; 95% CI: 1.67–3.80), which was higher than the risks observed for depression and anxiety.^52^ Psychotic disorders have been linked to accelerated aging and cognitive decline, which may potentially explain their greater contribution to dementia risk, compared with other psychiatric disorders.^52,55^

Climate change and population aging are two significant challenges that coexist in the 21st. The global exposure to floods is considerable,^6^ and projections indicate that flood hazards will increase across over half of the globe driven by global warming.^19^ Compared to younger adults, older people are more susceptible to both dementia and the ramifications of natural disasters, particularly those characterized by restricted mobility and preexisting health conditions. It is crucial to develop disaster management plans tailored to older people to reduce exposure levels and mitigate adverse health impacts. As suggested by our findings, targeted interventions to improve psychiatric conditions could be beneficial for dementia prevention in flood-affected areas. These efforts might include providing suitable residential relocation options, arranging mental health interventions; promoting social contact, and prioritizing older people for compensation, social and community support, and healthcare services. For example, evidence suggests that mental health interventions (e.g., psychotherapies, psychoeducation or training) administered by health professionals are effective in alleviating PTSD symptoms following natural disasters.^56^ Another example is the Daily Supportive Text Message Program, which delivers supportive and informative messages after disasters.^57^ This program has been shown to significantly reduce anxiety and stress levels during the COVID-19 pandemic and may serve as a population-level mental health intervention for individuals affected by natural disasters and other emergencies.^57^ Moreover, understanding the causal pathway linking floods to dementia can provide valuable insight into disease etiology and inform effective prevention strategies to reduce disease burden. Future research could benefit from our findings by considering psychiatric disorders when investigating the impacts of environmental exposures on dementia.

This study has several strengths. First, it benefits from a large population-based sample, with comprehensive assessments of demographic factors, lifestyles, and socioeconomic status. These covariates were adjusted in the model to capture the independent effect of floods on dementia. Second, unlike many previous studies that relied on questionnaire surveys or interviews to determine whether an individual was exposed to certain flood events, the present study used a flood index to measure long-term flooding exposure. By considering the duration, severity, and multiple exposures to floods based on participants’ home addresses, this approach reduces potential recall bias and provides a more accurate representation of the cumulative exposure to floods. Third, taking advantage of a follow-up period of nearly 13 years, we were able to establish the temporal sequence of events, ensuring that exposure precedes the occurrence of psychiatric disorders, which in turn precede the onset of dementia. Adhering to the fundamental assumptions of time order in causal inference adds rigor and enhances the validity of this study.

Some limitations should be acknowledged. Despite extensive adjustment for covariates, the presence of unmeasured confounders and exposure-induced mediator-outcome confounding factors cannot be completely ruled out. Although we linked flood events to participants’ home addresses, this does not provide a precise measure of individual-level exposure because participants may implement protective measures that reduce their risk. For example, building materials and structural features—critical factors for disaster resilience that can significantly reduce flood exposure—were not directly available in our data set.^58^ Instead, we used proxies such as socioeconomic status and regional deprivation to indirectly account for housing resilience. Future research should incorporate more detailed housing data to better assess the role of housing resilience in the health impacts of floods. Our participants were predominately white residents in the UK, and more likely to be healthier and wealthier, therefore, the generalizability of our findings to a broader population, particularly individuals from different ethnic groups and those in low- and middle-income countries, may be limited. Moreover, several covariates included in the model, such as lifestyles and socioeconomic status, were only measured once during the baseline survey. This lack of repeated measurements may lead to misclassification and not fully capture changes in socioeconomic status and behavioral lifestyles over time. Lastly, there is a possibility that individuals relocate away from the flood-affected region, before the arrival of floodwaters, and return after the floods have subsided. As we used participants’ home addresses to determine flooding exposure, there might be an overestimation of the actual level of exposure, resulting in the underestimation of the observed association.

## 5 Conclusions

In conclusion, our study indicates an increased risk of dementia associated with long-term exposure to floods, and psychiatric disorders may have a crucial mediating effect on flood-related dementia. These results contribute to a deeper understanding of the underlying disease mechanisms and have implications for the development of targeted prevention strategies to reduce the burden of dementia.

## Supplementary Material



## Data Availability

Data used in this study are available through registration on the UK Biobank (https://www.ukbiobank.ac.uk/).

## Notes

Baseline characteristics of study participants excluded missing values; Flood severity definition; Spatial distribution of flooding exposure; regression results stratified by each subtype of psychiatric disorders; Results from sensitivity analyses (PDF)
