# Supplementary material for: Psychiatric Disorders Mediate the Association between Floods and Dementia: A Prospective Cohort Study in the UK Biobank
Source: Environ Health (Wash). 2025 Mar 13;3(6):680–9. doi: 10.1021/envhealth.4c00241 (PMC12186207; doi:10.1021/envhealth.4c00241)
Supplement: Supplementary file 1 [file eh4c00241_si_001.pdf]

Supporting Information

Psychiatric disorders mediate the association between floods and dementia: a prospective cohort study in the UK Biobank

Yao Wu[1], Bo Wen[1], Danijela Gasevic[1,2], Rongbin Xu[1], Zhengyu Yang[1], Pei Yu[1], Yanming Liu[1], Guowei Zhou[3], Yan Zhang[3], Jiangning Song[4], Hong Liu[3,a], Shanshan Li[1,a], Yuming Guo[1,a,*]

Affiliations:

[1] School of Public Health and Preventive Medicine, Monash University, Melbourne, VIC, 3004, Australia;

[2] Centre for Global Health, Usher Institute, The University of Edinburgh, Teviot Place, Edinburgh EH8 9AG, United Kingdom;

[3] Department of Dermatology, Xiangya Hospital, Central South University, Changsha, Hunan, 410008, China;

[4] Monash Biomedicine Discovery Institute, Department of Biochemistry and Molecular Biology, Monash University, Melbourne, VIC, 3800, Australia.

[a] Co-senior authors

***Corresponding author:** Professor Yuming Guo, School of Public Health and Preventive Medicine, Monash University, Level 2, 553 St Kilda Road, Melbourne, VIC, 3004, Australia. Tel: +61 3 9905 6100. E-Mail: yuming.guo@monash.edu.

Table of Contents

Table S1. Baseline characteristics of study participants who were included and excluded due to missing values.
Table S2. Definitions of different severities of flood events.
Table S3. Summary statistics of annual flood index.
Figure S1. The spatial distribution of the flooding exposure over different exposure windows.
Figure S2. Associations between incident dementia and floods, stratified by depression.
Figure S3. Associations between incident dementia and floods, stratified by anxiety.
Figure S4. Associations between incident dementia and floods, stratified by substance misuse.
Figure S5. Associations between incident dementia and floods, stratified by stress-related disorders.
Figure S6. Associations between incident dementia and floods, stratified by psychotic disorders.
Table S4. Association of incident dementia with floods stratified by psychiatric disorders, with and without adjustment for mean temperature and relative humidity.
Table S5. Association of incident dementia with floods stratified by psychiatric disorders, with and without adjustment for other chronic diseases.
Table S6. Association of incident dementia with floods stratified by psychiatric disorders, comparing all participants' analyses with analyses among participants living in the current address for at least ten years.
Table S7. Association of incident dementia with floods stratified by psychiatric disorders, comparing the complete case analyses with the multiple imputation analyses.
Table S8. Bootstrap results for total, direct, and indirect effects of floods on incident dementia through psychiatric disorders by using varying time intervals between the mediator and outcome.
Table S9. Association of incident dementia with floods stratified by psychiatric disorders, with and without adjustment for apolipoprotein E status.
Table S10. Association of incident dementia with floods stratified by psychiatric disorders, with and without adjustment for social isolation.

Table S1. Baseline characteristics of study participants included and excluded due to missing values.

	Participants excluded due to missing values	Participants included
N	200,000	227,033
Age, mean (SD)	57.21 (8.14)	56.05 (8.06)
Male (%)	75337 (37.9)	122171 (53.8)
White ethnicity (%）	179140 (91.1)	220749 (97.2)
BMI, mean (SD)	27.76 (5.10)	26.94 (4.29)
Household income (%)		
<£18,000	40141 (30.0)	35077 (15.5)
£18,000-30,999	37312 (27.9)	53853 (23.7)
£31,000-51,999	32386 (24.2)	63537 (28.0)
£52,000-100,000	19861 (14.9)	57423 (25.3)
>£100,000	3966 (3.0)	17143 (7.6)
Education attainment (%)		
Degree	44117 (23.2)	24839 (10.9)
A	34983 (18.4)	35078 (15.5)
O	61940 (32.6)	75679 (33.3)
None of the above	49041 (25.8)	91437 (40.3)
Smoking (%)		
Never	118753 (60.4)	124148 (54.7)
Previous	60648 (30.9)	84744 (37.3)
Current	17073 (8.7)	18141 (8.0)
Non-moderate alcohol consumer	(%) 68453 (78.4)	179918 (79.2)
Healthy diet score, mean (SD)	0.58 (0.49)	0.56 (0.50)
Physical activity (%)		
Low	23200 (20.1)	38935 (17.1)
Middle	27963 (24.2)	58691 (25.9)
High	64304 (55.7)	129407 (57.0)
High Townsend deprivation index, n (%)	104314 (52.6)	103042 (45.4)
Psychiatric disorders (%)	23505 (11.8)	21629 (9.5)
Dementia (%)	3160 (1.6)	2028 (0.9)

Table S2. Definitions of different severities of flood events.

Severity class	Value	Definition
No flood events	0	No flood events
Large flood events	1	Significant damage to structures or agriculture; fatalities; and/or 1–2 decades-long reported interval since the last similar event.
Very large events	1.5	With an estimated recurrence interval greater than 2 decades but less than 100-year, and/or with a local recurrence interval of 1–2 decades and affecting a large geographic region (> 5000 km^2).
Extreme events	2	With an estimated recurrence interval greater than 100 years.

Table S3. Summary statistics of annual flood index.

Exposure windows	Without dementia			With dementia		
	Mean (Standard deviation)	Median (Interquartile range)	Range	Mean (Standard deviation)	Median (Interquartile range)	Range
Lag 0	16.8 (19.5)	5.0 (0.0, 36.0)	0.0-51.0	18.7 (20.1)	11.0 (0.0, 45.0)	0.0-48.0
Lag 0-1	15.4 (14.7)	8.0 (2.5, 24.0)	0.0-49.5	16.1 (15.0)	8.0 (2.5, 24.0)	0.0-46.5
Lag 0-2	12.9 (12.6)	5.3 (1.7, 20.5)	0.0-48.0	13.1 (12.7)	6.7 (1.7, 20.5)	0.0-48.0
Lag 0-3	10.1 (10.2)	4.0 (1.9, 15.4)	0.0-36.0	10.2 (10.0)	5.0 (1.9, 15.4)	0.0-36.0
Lag 0-4	8.1 (8.1)	4.2 (1.5, 12.3)	0.0-28.8	8.2 (8.0)	4.2 (1.5, 12.3)	0.0-28.8
Lag 0-5	6.9 (6.7)	3.5 (1.8, 10.2)	0.0-24.0	6.9 (6.6)	3.5 (1.8, 10.2)	0.0-24.0
Lag 0-6	6.1 (5.8)	3.1 (1.6, 9.2)	0.0-21.4	6.1 (5.7)	3.1 (1.6, 9.2)	0.0-21.4
Lag 0-7	5.5 (5.1)	3.1 (1.5, 8.1)	0.0-18.8	5.5 (5.0)	3.1 (1.5, 8.1)	0.0-18.8

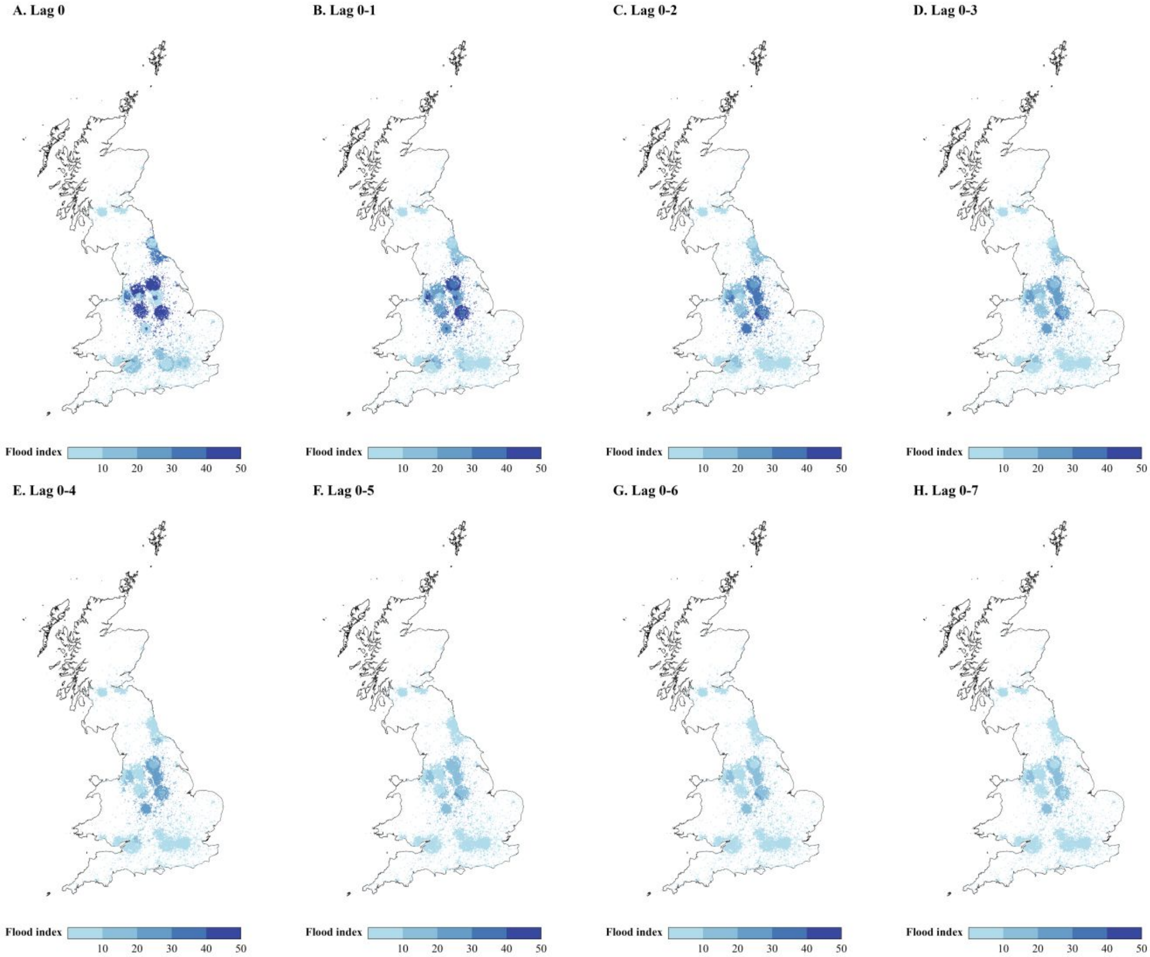

Figure S1. The spatial distribution of the flooding exposure over different exposure windows.

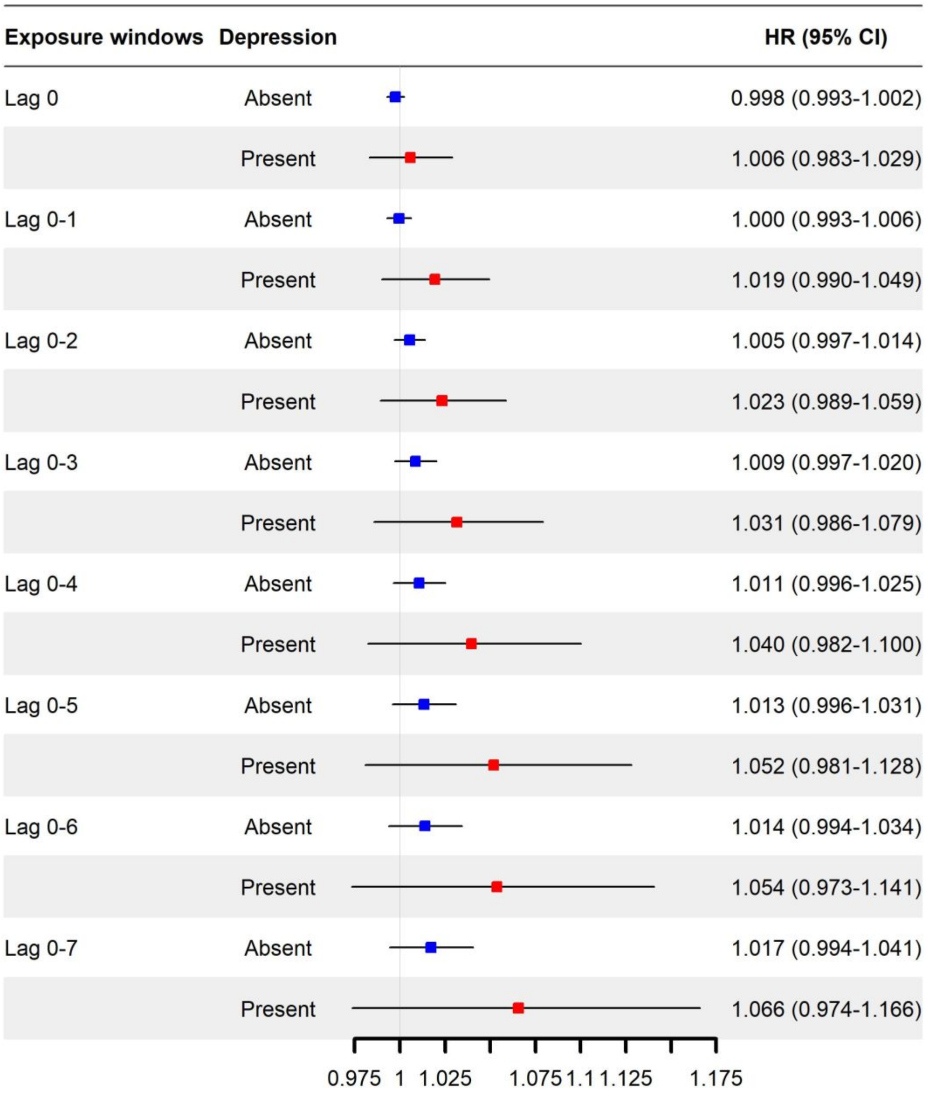

Figure S2. Associations between incident dementia and floods, stratified by depression. Estimates are hazard ratios associated with per unit increase in flood index derived from Cox proportional hazards regression models. Models are adjusted for age, body mass index, sex, ethnicity, annual household income before tax, educational attainment, smoking status, drinking status, physical activity, and assessment centre.

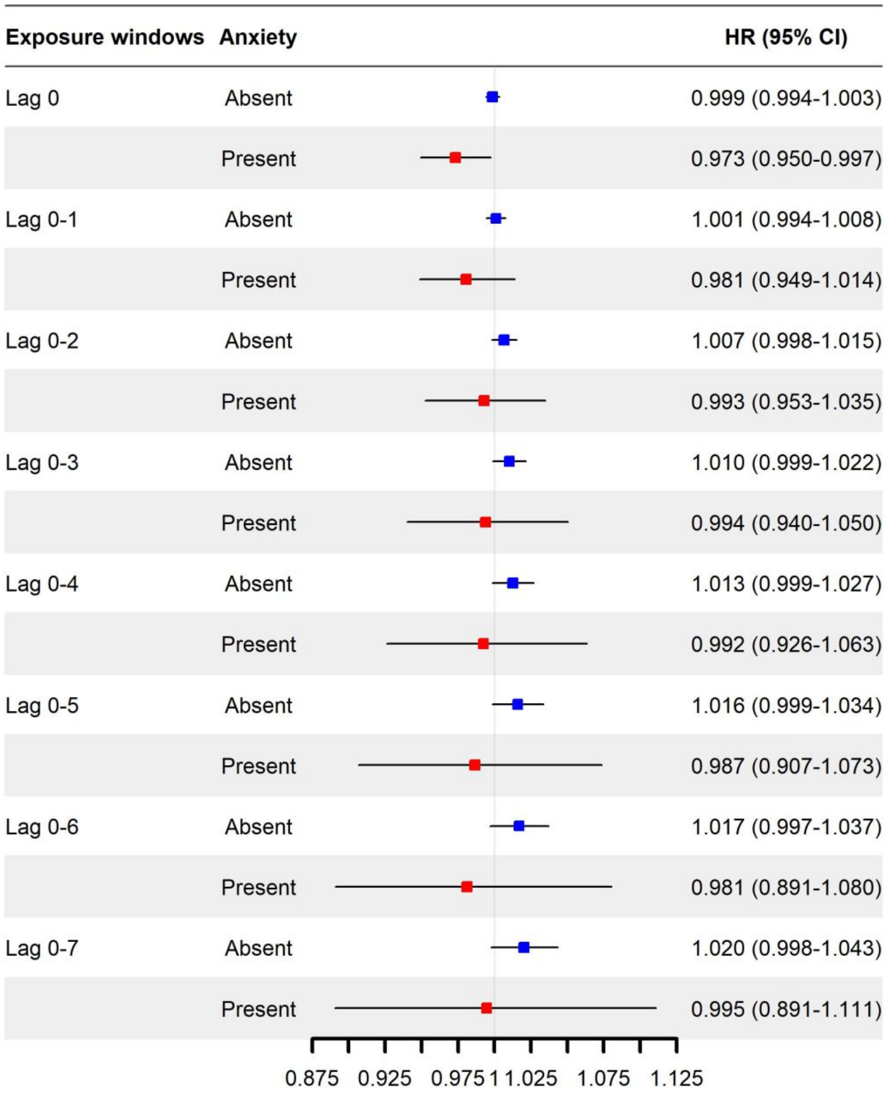

Figure S3. Associations between incident dementia and floods, stratified by anxiety. Estimates are hazard ratios associated with per unit increase in flood index derived from Cox proportional hazards regression models. Models are adjusted for age, body mass index, sex, ethnicity, annual household income before tax, educational attainment, smoking status, drinking status, physical activity, and assessment centre.

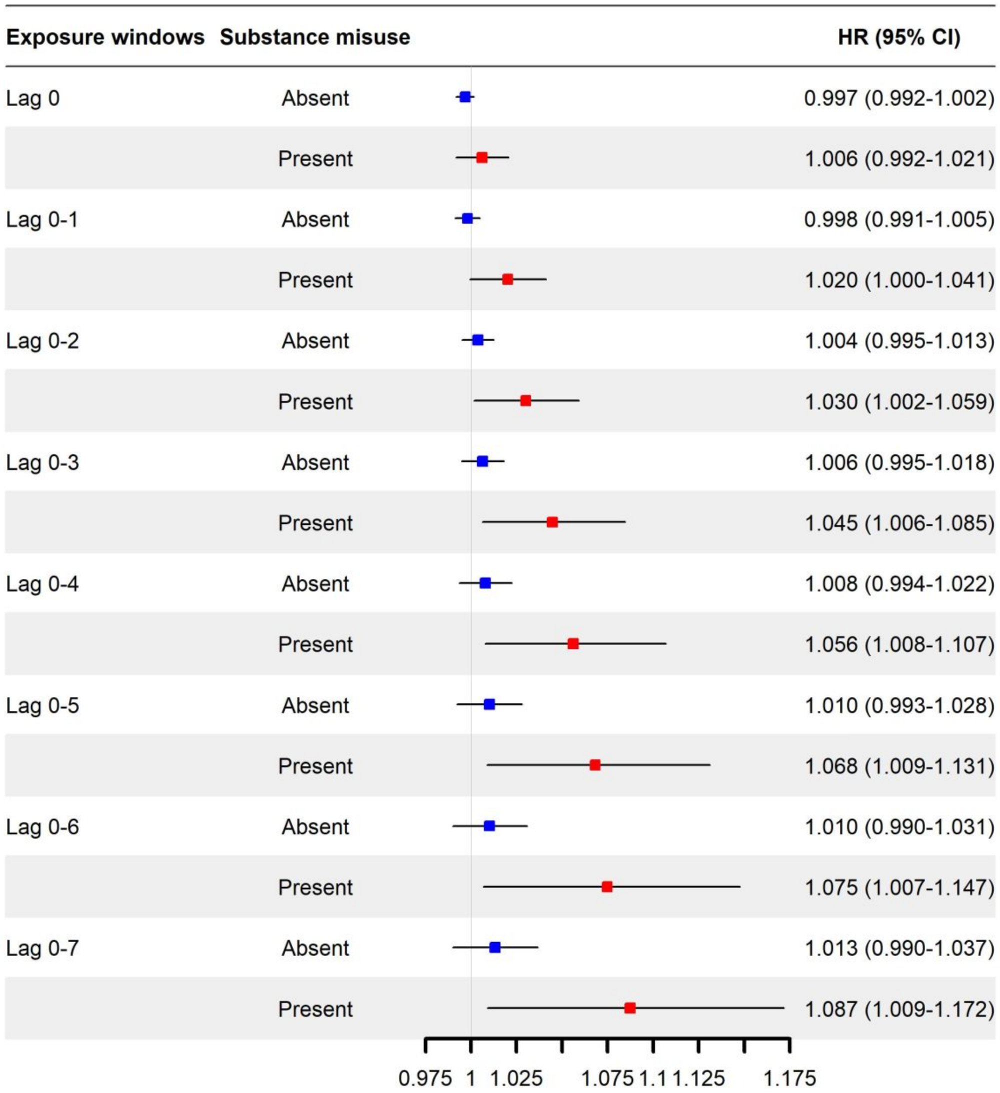

Figure S4. Associations between incident dementia and floods, stratified by substance misuse. Estimates are hazard ratios associated with per unit increase in flood index derived from Cox proportional hazards regression models. Models are adjusted for age, body mass index, sex, ethnicity, annual household income before tax, educational attainment, smoking status, drinking status, physical activity, and assessment centre.

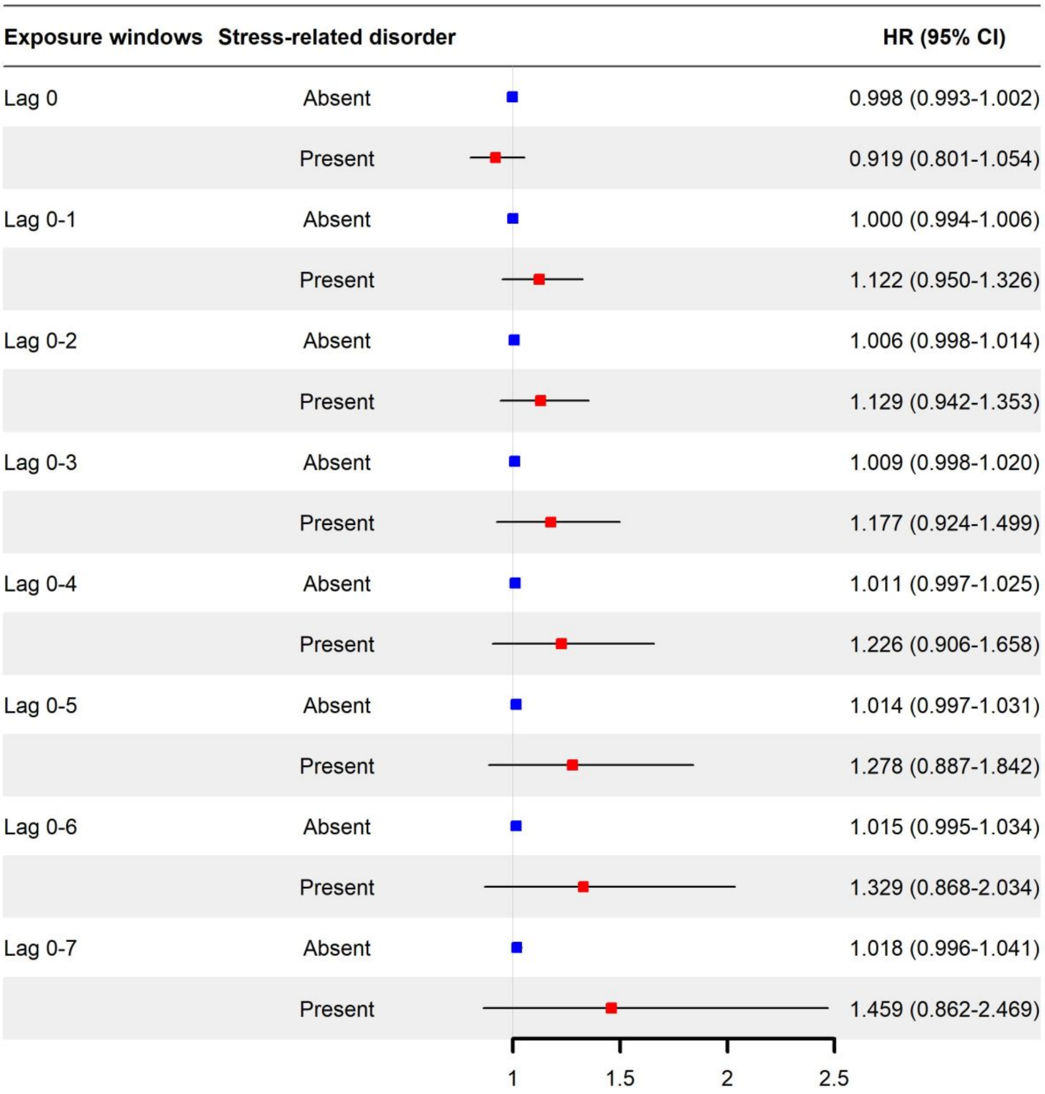

Figure S5. Associations between incident dementia and floods, stratified by stress-related disorders. Estimates are hazard ratios associated with per unit increase in flood index derived from Cox proportional hazards regression models. Models are adjusted for age, body mass index, sex, ethnicity, annual household income before tax, educational attainment, smoking status, drinking status, physical activity, and assessment centre.

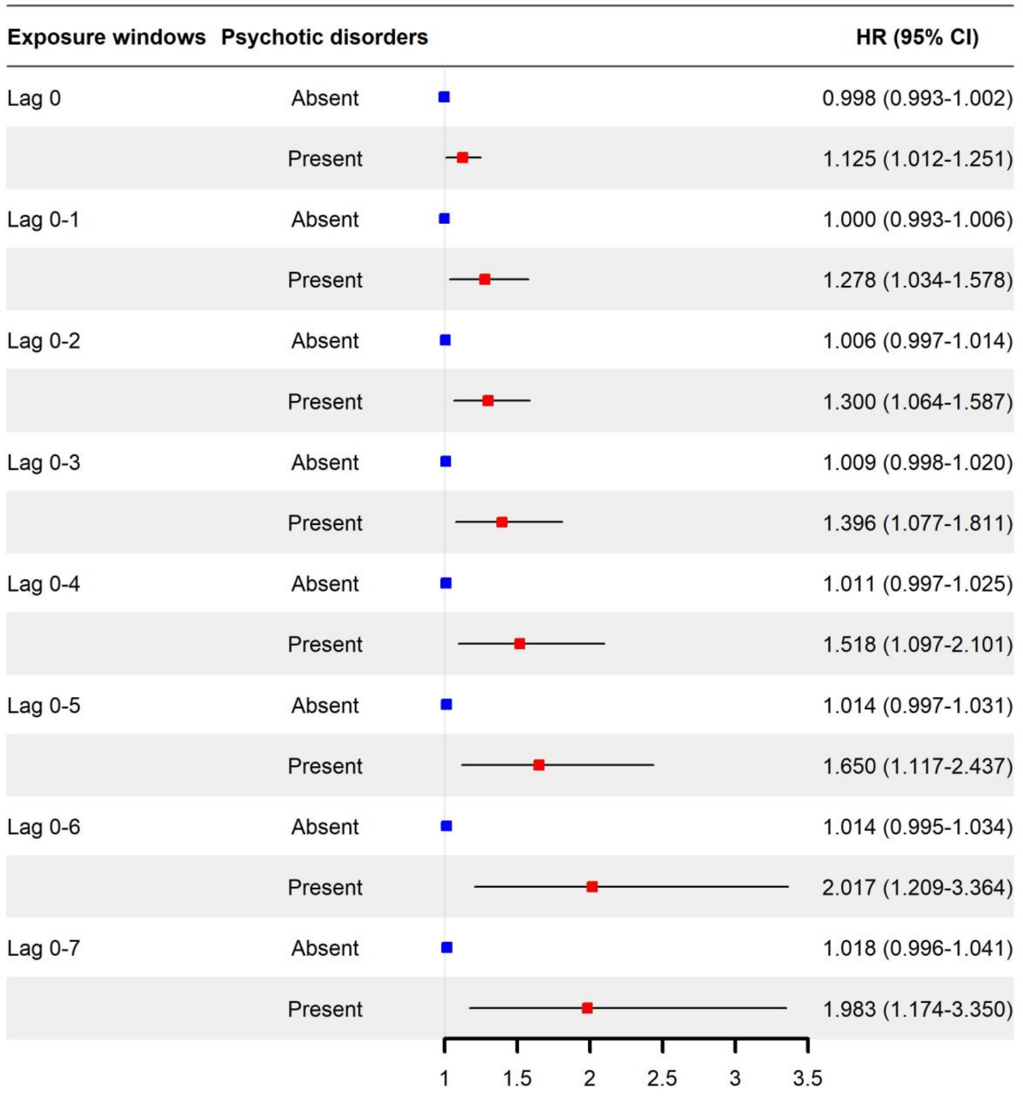

Figure S6. Associations between incident dementia and floods, stratified by psychotic disorders. Estimates are hazard ratios associated with per unit increase in flood index derived from Cox proportional hazards regression models. Models are adjusted for age, body mass index, sex, ethnicity, annual household income before tax, educational attainment, smoking status, drinking status, physical activity, and assessment centre.

Table S4. Association of incident dementia with floods stratified by psychiatric disorders, with and without adjustment for mean temperature and relative humidity.

Psychiatric disorders	Adjusted for temperature and relative humidity	HR (95% CI)
Absent	Yes	1.008 (0.983-1.033)
Absent	No	1.009 (0.986-1.032)
Present	Yes	1.047 (1.006-1.089)
Present	No	1.042 (1.004-1.081)

Estimates are hazard ratios associated with per unit increase in flood index derived from Cox proportional hazards regression models. Models are adjusted for age, body mass index, sex, ethnicity, annual household income before tax, educational attainment, smoking status, drinking status, physical activity, assessment centre, temperature, and relative humidity.

Table S5. Association of incident dementia with floods stratified by psychiatric disorders, with and without adjustment for other chronic diseases.

Psychiatric disorders	Adjusted for other chronic diseases [a]	HR (95% CI)
Absent	Yes	1.006 (0.983-1.030)
Absent	No	1.009 (0.986-1.032)
Present	Yes	1.042 (1.004-1.081)
Present	No	1.042 (1.004-1.081)

Estimates are hazard ratios associated with per unit increase in flood index derived from Cox proportional hazards regression models. Models are adjusted for age, body mass index, sex, ethnicity, annual household income before tax, educational attainment, smoking status, drinking status, physical activity, and assessment centre.

[a] Other chronic diseases include hypertension, diabetes mellitus. cardiovascular diseases, and chronic obstructive pulmonary disease.

Table S6. Association of incident dementia with floods stratified by psychiatric disorders, comparing all participants' analyses with analyses among participants living in the current address for at least ten years.

Psychiatric disorders	Group	HR (95% CI)
Absent	All participants	1.009 (0.986-1.032)
Absent	Participants living in the same location ≥ 10 years	1.006 (0.978-1.034)
Present	All participants	1.042 (1.005-1.081)
Present	Participants living in the same location ≥ 10 years	1.060 (1.012-1.112)

Estimates are hazard ratios associated with per unit increase in flood index derived from Cox proportional hazards regression models. Models are adjusted for age, body mass index, sex, ethnicity, annual household income before tax, educational attainment, smoking status, drinking status, physical activity, and assessment centre.

Table S7. Association of incident dementia with floods stratified by psychiatric disorders, comparing the complete case analyses with the multiple imputation analyses.

Psychiatric disorders	Dataset	HR (95% CI)
Absent	Imputed	1.003 (1.003-1.003)
Absent	Original	1.006 (0.983-1.029)
Present	Imputed	1.011 (1.011-1.012)
Present	Original	1.039 (1.002-1.078)

Estimates are hazard ratios associated with per unit increase in flood index derived from Cox proportional hazards regression models. Models are adjusted for age, body mass index, sex, ethnicity, annual household income before tax, educational attainment, smoking status, drinking status, physical activity, and assessment centre.

Table S8. Bootstrap results for total, direct, and indirect effects of floods on incident dementia through psychiatric disorders by using varying time intervals between the mediator and outcome.

Time difference between outcome and mediators	Indirect effect (95% CI)	Direct effect (95% CI)	Total direct (95% CI)	Proportion (%)
1 year	1.067 (1.021, 1.122)	1.018 (1.007, 1.029)	1.087 (1.028, 1.155)	77.74
2 years	1.060 (1.018, 1.108)	1.018 (1.005, 1.030)	1.080 (1.023, 1.141)	75.71
3 years	1.048 (1.017, 1.087)	1.017 (1.006, 1.028)	1.066 (1.023, 1.118)	73.35

Estimates are hazard ratios associated with per unit increase in flood index derived from Cox proportional hazards regression models. 95% confidence intervals were computed using bootstrapping procedures (1000 replications). Models are adjusted for age, body mass index, sex, ethnicity, annual household income before tax, educational attainment, smoking status, drinking status, physical activity, and assessment centre. IE=IE1×IE2. TE=IE+DE. Abbreviations: IE, indirect effect. DE, direct effect. TE, total effect.

Table S9. Association of incident dementia with floods stratified by psychiatric disorders, with and without adjustment for apolipoprotein E status.

Psychiatric disorders	Adjusted for apolipoprotein E status	HR (95% CI)
Absent	Yes	1.006 (0.983-1.029)
Absent	No	1.006 (0.983-1.029)
Present	Yes	1.069 (1.011-1.131)
Present	No	1.070 (1.012-1.131)

Estimates are hazard ratios associated with per unit increase in flood index derived from Cox proportional hazards regression models. Models are adjusted for age, body mass index, sex, ethnicity, annual household income before tax, educational attainment, smoking status, drinking status, physical activity, assessment centre, and apolipoprotein E status.

Table S10. Association of incident dementia with floods stratified by psychiatric disorders, with and without adjustment for social isolation.

Psychiatric disorders	Adjusted for social isolation	HR (95% CI)
Absent	Yes	1.003 (1.003-1.003)
Absent	No	1.006 (0.983-1.029)
Present	Yes	1.011 (1.011-1.012)
Present	No	1.039 (1.002-1.078)

Estimates are hazard ratios associated with per unit increase in flood index derived from Cox proportional hazards regression models. Models are adjusted for age, body mass index, sex, ethnicity, annual household income before tax, educational attainment, smoking status, drinking status, physical activity, and assessment centre.
